# A Qualitative Exploratory Study of Patient Safety and Nurses’ Body Awareness Using Motion‐Capture‐Linked Avatars

**DOI:** 10.1155/jonm/9415472

**Published:** 2026-03-26

**Authors:** Chieko Fujii

**Affiliations:** ^1^ Faculty of Nursing and Medical Care, Keio University, Fujisawa, Japan, keio.ac.jp

**Keywords:** avatar, body awareness, nontechnical skills, patient safety, privacy

## Abstract

This study aimed to explore the ongoing possibilities of motion‐capture‐linked avatars in patient safety education, with the goal of enhancing nurses’ body awareness, thereby improving patient safety and nontechnical skills. Six nurses with an average experience of 29.0 ± 4.8 years participated in this pilot, exploratory qualitative study. Actual situations using avatars were initially demonstrated through video. Participants subsequently wore mobile motion‐capture devices, with responses obtained through interviews and questionnaires based on their experience using the avatars. Qualitative content analysis was conducted, and the responses were organized into the following three themes: usability of the avatars as a new technology, promoting body awareness, and patient safety using avatars. The findings showed that avatars enabled nurses to learn about body movements without the need to identify specific individuals, while protecting their privacy and surroundings. However, using avatars alone was insufficient to fully convey the situation. Verbalizing the avatar’s movements was also found to be important. This study indicates that motion capture and avatars can contribute to new patient safety education through physical awareness when employed in nursing situations. The movement‐based knowledge sharing gained through this new approach (avatars) can contribute to improving nursing management quality.

## 1. Introduction

Nursing management is witnessing the emergence of various management strategies. However, nurses rarely have the opportunity to examine their own body movements. If motion capture and avatars are used to enhance nurses’ body awareness, the possibilities for improving patient safety management may expand.

Nurses engage in various forms of communication with patients through nonverbal interactions. Interactions between nursing professionals and individuals involved in the care process entail the exchange of emotions, actions, and experiences [1]. Patients usually perceive care through nurses’ body language, as the body communicates even in the absence of words. Elements such as listening, touch, body language, eye contact, a positive demeanor, and helping behaviors demonstrated by small acts of kindness that go beyond routine healthcare and indicate empathic/compassionate encounters are also important in nursing [2]. Therefore, healthcare professionals should continually develop their verbal and nonverbal communication, empathy, active listening, and collaboration skills [3]. Research on early rehabilitation in intensive care units has emphasized the importance of integrated physical communication, combining verbal and physical prompts, to encourage patient participation [4]. Despite its significance, the potential of learning body language is usually overlooked in education [5].

Although nurses and patients respond to each other’s movements, nurses have few opportunities to become aware of their own body movements. Self‐awareness is essential for nurses to improve relationships with and care for patients [6]. Some studies in nursing education employed a dance movement therapy workshop to enhance undergraduate nursing students’ relational and nonverbal communication skills [7]. Body pedagogics can reassert the central role of embodiment in medical education and reveal unusual habits and disregarded aspects of embodiment, enabling them to be considered as instructional strategies and subjects of research and evaluation [8].

Nurses perceive the emotions and pain of their patients. Observing somatic pain, such as witnessing the body pain of others, activates brain regions involved in negative emotion processing and neuronal structures engaged in somatosensation and skeletal muscle control [9]. Based on existing theories and research, Monin and Schulz [10] proposed that caregivers may experience similar, complementary, and/or defensive emotions in response to care recipients’ problems through mechanisms such as cognitive empathy, mimicry, and conditioned learning, placing them at risk of psychological and physical morbidity. Emotions serve as natural vectors for interacting with others and are expressed through human behavior, perception, and bodily functions [11]. However, nurses rarely have the opportunity to realize how patients’ movements affect their own responses.

Nontechnical skills—defined as cognitive and social skills used by individuals and teams to reduce error and improve performance in complex systems—are increasingly recognized as key contributors to patient safety [12]. Human factors are also an important component of the nontechnical skills that contribute to patient safety. Nurses should recognize their own and/or patients’ movements and subsequently observe and anticipate the patient’s actions. However, current evidence regarding body awareness, patient movement, and their interactions remains limited. Nurses can better understand their physical awareness and patient movements by focusing on the human element of bodily sensation, thereby ensuring greater patient safety.

Currently, body movements can be determined in a few ways, with the use of video as one emerging approach. However, individuals are generally concerned about their own body shape and clothing when on video. The privacy of others, such as patients’ appearance at the bedside, should be respected. Failure to respect patient privacy can undermine trust in the patient–nurse relationship [13]. Health institutions face numerous challenges in maintaining confidentiality and preserving patient information amid recent advances in technology. Patient privacy may also possess connotative cultural and religious values. Typically, a patient’s body is not expected to be overly exposed, with only the area requiring intervention uncovered. It is immediately covered after the intervention [14]. Nurses have limited opportunities to directly observe patients or exchange interpretations of a patient’s situation. Since privacy is crucial, restrictions are usually placed on filming the movements of both nurses and patients. Against this backdrop, nursing is considered a field where education and research on human movement awareness and its optimization remain underdeveloped.

Modern medical pedagogical strategies are shifting toward virtual patient simulations [15]. Virtual clinical simulation in nursing education is an innovative teaching and learning strategy that provides immersive self‐regulated training in nursing practice, reproducing real‐life experiences and feedback in a safe, interactive, dynamic, and enjoyable virtual environment [16]. Several studies have described simulation debriefing as an integral part of the simulation process for clinical teaching and learning in nursing education. It is highly valued during nursing students’ transition from academics to clinical practice [17].

Visualization using personalized avatars may help nurses understand their individual roles in population health [18]. Although avatars have demonstrated effectiveness, barriers may arise if nurses are unaware of their benefits and/or reluctant to adopt new technology perceived as replacing aspects of human care, such as nursing. Gaining an understanding of how such technologies function can be useful for patient safety; however, their potential use in safety education remains unclear [19].

Products that enable three‐dimensional (3D) full‐body tracking, such as those used for dancing, are currently accessible. They eliminate an individual’s concern about being recorded and help protect privacy when linked to an avatar. In the future, avatars combined with mobile motion capture may be used to support patient safety based on evidence of body awareness. However, no exploratory research has investigated this new form of body awareness.

This study was based on the hypothesis that nurses have limited opportunities to develop awareness of their own body movements and lack the necessary training to comprehensively describe their own and/or patients’ movements. Using avatars combined with motion capture can increase nurses’ awareness of their physical movements, thereby contributing to improved patient safety. Therefore, this exploratory study aimed to assess the feasibility of this approach and obtain preliminary insights.

## 2. Materials and Methods

### 2.1. Study Design

This study employed a qualitative exploratory design and used content analysis based on example videos presented using avatars with mobile motion capture, alongside questionnaire responses that mirrored the content of postexperience interviews. A qualitative content analysis using the inductive approach of Elo and Kyngäs [20] was conducted, adhering to the Consolidated Criteria for Reporting Qualitative Research checklist.

### 2.2. Participants

The importance of patient safety gained widespread recognition in Japan after two serious medical accidents in 1999 [21]. One involved surgeries performed on two patients undergoing lung and heart treatments, who were mistaken for each other. The other case comprised a patient who died after disinfectant was mistakenly injected into an intravenous drip instead of the requisite medication. These incidents marked a major turning point, prompting a shift in the medical field toward creating an environment that prevents medical accidents, based on the premise that “anyone can make mistakes.” Many hospitals are establishing independent patient safety departments to which nurses are assigned. Nurses who assume these roles typically have extensive nursing experience although this experience may vary. They are also highly trusted by nursing managers.

This study targeted nurses with patient safety management experience, reflecting a perspective relevant to improving patient safety education. The following inclusion criteria were applied for participant recruitment: (i) nurses, (ii) experience in patient safety management, and (iii) experience in in‐hospital safety education.

Participants were recruited to ensure diversity by sending letters to all directors of nursing at 19 hospitals with ≥ 200 beds located in the same or nearby area as the author’s office. The patient safety group of the local nursing association was also invited to participate. Directors of nursing at two hospitals responded, confirming that two nurses could participate in the study. Two more nurses from the same hospital, belonging to the patient safety group of the local nursing association, were also able to participate. Nurses who wished to participate were provided with a private, easily accessible, private conference room, where they could discuss their concerns without being overheard. Participants were notified of the location and start time by email and in writing and were reminded that participation was entirely voluntary.

### 2.3. Author’s Positionality

The author, a university professor with a PhD and experience in qualitative and quantitative research, developed the interview guide and conducted the interviews. A Japanese nurse with over 25 years of experience in nursing education and prior experience as a lecturer in information science. Data were obtained through semistructured individual interviews and subsequently analyzed. Specifically, the author has researched patient safety but has never worked in a hospital patient safety office. Participants were selected without prior knowledge of their work environment or experiences. No hierarchical or vested interest existed between the author and participants. Participants perceived the author as someone who had not shared their experiences and described their situations in their responses. The researcher listened carefully to the participants’ situations to eliminate potential bias. While the focal topic of patient safety was shared with participants, conducting the avatar experience initially helped prevent leading responses, allowing participants to address the topic voluntarily.

### 2.4. Research Procedure

#### 2.4.1. Creating an Avatar

**FIGURE 2:**
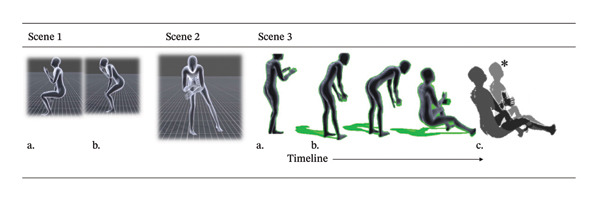
Example of a scene using motion‐capture‐linked avatars. Scene 1: Getting up from a chair: (a) sitting and (b) getting up. Scene 2: Assistance with getting up from a chair. Scene 3: (a) Standing behind an older woman to help her walk. (b) Unable to maintain posture. (c) She sat on the caregiver. ^∗^Added an image of an older woman.

The “mocopi” (Sony Corporation; https://www.sony.jp/mocopi/) is a mobile motion‐capture device whose sensors are attached to the back of the head, lower back, both wrists, and both ankles. It enables 3D full‐body tracking, regardless of location, and can be instantly converted into an avatar using specific software. With the cooperation of a volunteer, the author filmed videos of caregiving situations wearing “mocopi” in advance (Figures 1 and 2). A sample video of nursing situations with the researcher’s avatar was provided. Both the “mocopi” and real‐life videos contained two scenes. Each scene comprises a 2‐min recorded video. Participants were requested to view these on a computer at the beginning of the survey (see Appendix 1).

FIGURE 1Contrast using motion‐capture‐linked avatars and real‐life examples. (a) Avatar using mocopi, Sony Corporation. (b) Real‐life examples.

**Figure:**
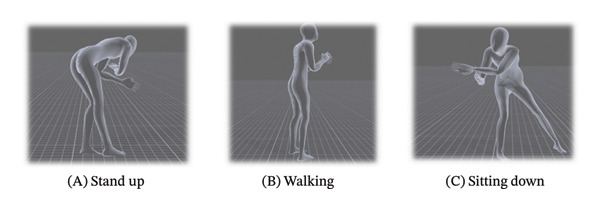
(a)

**Figure:**
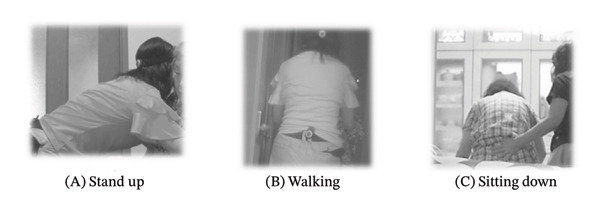
(b)

Following this, participants experienced the use of “mocopi” and transformed their appearance using avatars, as illustrated in Figure 1. They were also allowed to become one of the pre‐existing animated female avatars. Responses were deidentified and analyzed, and participants received a USB containing a video of their avatar as a token of appreciation.

#### 2.4.2. Basic Resources

For this study, videos of caregiving situations were recorded for presentation during the interviews. The scenarios were also filmed with the participant wearing the “mocopi.” Video editing software (EDIUS10; Grass Valley K.K., Japan) was used to remove backgrounds, eliminate unnecessary footage, and extract still images.

During the avatar experience before the interview, participants wore the “mocopi” and were shown their movements transformed into an avatar using an application preinstalled on an iPad Mini (5th generation). The interview, including the avatar experience, lasted approximately 60 min. Expenses included the purchase of the “mocopi” and the use of a conference room with wireless LAN.

#### 2.4.3. Interview and Questionnaire Items

Questions were asked based on the interview guide (see Appendix 1). The questionnaire collected data on participants’ age, years of nursing experience, sex, and prior experience using an avatar. Data from six different individuals were summarized by calculating the mean and standard deviation of numerical responses using Microsoft Excel.

### 2.5. Ethical Considerations

This study adhered to the Declaration of Helsinki and was approved by the Ethics Committee of Keio University, Faculty of Nursing and Medical Care (approval number: 334). Participants were informed of their rights, the ethical guidelines, data confidentiality, and the study objectives. Participation was voluntary, and participants could withdraw at any time without repercussion. They were assigned numbers to ensure anonymity for recorded interviews and questionnaires. Written informed consent was obtained from all six participants.

### 2.6. Data Collection

A separate questionnaire was administered to obtain individual opinions since the group interview included two participants. The questions asked were “How is the usability of the avatar?” “Can we promote body awareness?” and “Can we use educational materials to learn hazard prediction?”

To facilitate the interview process, participants received materials approved by the ethics committee outlining the study’s purpose and objectives. Permission was obtained from the participants to audio‐record the avatar experience and interviews. Data were obtained between August and September 2024, with each group interview lasting approximately 90 min.

The two nurses from each facility were of different ages. Although the participants performed the same role, their nursing experience varied. They responded to interview questions prepared by the researcher and discussed various patient safety issues, including falls. Categories were established during the second round. Data saturation was reached after the third interview, as no new categories emerged in this study.

The study began with the researcher’s self‐introduction. All six female nurses completed the interviews and questionnaires. The participants’ mean age and average number of years of nursing experience were 50.8 ± 5.1 and 29.0 ± 4.8 years, respectively. One participant had experience using avatars. No inconsistencies or unclear statements were identified. Therefore, participants were not asked to review their responses.

### 2.7. Analysis Method

The analysis was conducted using the five‐step method of Granhiem and Landman [22], with additional reference to the content analysis methods of Kim [23] and Ashghali‐Farahani [24].

These steps were as follows: (i) each interview was transcribed immediately after it was conducted, (ii) all interviews were read to gain familiarity with the data and an overall understanding of the topic, (iii) units and major codes were identified, (iv) codes were categorized, and (v) major themes were extracted.

An inductive approach was applied to coding the data. No analysis software was used since the data comprised simple sentences. Identification was performed through manual coding. Codes were integrated and categorized based on similarities. The coding was conducted by one researcher.

### 2.8. Ensuring the Validity of Research Results

The criteria of Lincoln and Guba [25] were applied to ensure credibility, transferability, dependability, and confirmability. Credibility was established by selecting participants with varied experiences. Interviews, questionnaires, and observations were conducted for triangulation purposes. Transferability was achieved by accurately describing participants, sampling method, and the time and place of data collection. The materials presented to participants comprised the same preprepared videos. Dependability was achieved through an interview guide prepared for data collection. The interviews were conducted with participants’ consent while maintaining their privacy, allowing for candid feedback. All interviews were audio taped and transcribed to evaluate the findings’ dependability. Confirmability was achieved through a comprehensive description of the data, allowing evaluation via an external observer and gaining a clear understanding of the research process.

### 2.9. Limitations

Data saturation was considered to have been reached, as the responses obtained addressed the study’s hypothetical framework. Responses included the questions of this hypothesis such as the feasibility of using avatars in clinical practice; opinions on filming patients, nurses, and themselves; and the need for educational materials for medical safety and risk prediction. The uniformity and sample size were low; however, no negative opinions were observed regarding the use of avatars, and certain limitations in their use were identified.

## 3. Results

### 3.1. Participants’ Responses

Two participants were recruited from each of the three hospitals and were treated as pairs. In this study, participants initially watched a video of a created avatar and subsequently experienced it for themselves. After a discussion on nurses’ body awareness, all participants provided feedback on whether it could be used with patients and expressed a desire to learn about the interactions between patients and nurses. Nurses noted that explaining the situation using an avatar alone would be challenging. Most of the comments focused on the avatar’s potential, while the profound emotions that accompany a medical accident were never expressed.

The author labeled the answers according to their content, and responses were thematically analyzed and classified into three categories and subcategories. Specifically, the three categories were organized into the following themes: expression by avatar, protecting privacy and promoting body awareness, and patient safety using avatars. As a distinct case, an opinion addressed fall prevention (Table 1). Only a few in‐depth discussions occurred based on individual experiences since the theme centered on the potential use of avatars, preventing the creation of additional categories.

**TABLE 1:** Categories and subcategories: extracted answers from questionnaires only.

Category	Sub‐category
Usability of the avatars as a new technology	1. Learn movement
	2. Simplicity and ease of use
	3. Enjoyment
	4. Privacy protection

Promoting body awareness	1. Novice nurse training
	2. Used for patient guidance
	Promoting patient understanding
	Fall prevention
	3. Moves have not been revealed until now

Patient safety using avatars	1. Limitations of this avatar
	2. For risk prediction training

### 3.2. Usability of the Avatars as a New Technology

Responses were grouped into four categories as follows: learning movement, simplicity and ease of use, enjoyment, and privacy protection. Although some nurses are generally resistant to technology such as avatars, the responses in this study were positive. Some typical responses are as follows.

#### 3.2.1. Learn Movement

• I thought it would be great because you would be able to see people’s movements in real time (No. 2).
• We usually do not have the opportunity to observe our own movements objectively, do we? Even if we look in the mirror, our eyes inevitably go to the mirror, making it appear unnatural. It could be used for everyday movements since there is no opportunity to observe your own movements through video or other means; it is surprisingly difficult to determine whether you are performing the exercise safely (No. 5).

#### 3.2.2. Simplicity and Ease of Use

• I think the patients would be much happier with characters. It is easy and simple to become an avatar (No. 1).
• The movements are easy to understand. You can see movements such as standing up from a chair, and even perform such movements (No. 3).

#### 3.2.3. Enjoyment

• Normally, I would not do a pose like this. It is fun to become a different person (while becoming an animated girl). I will try turning around (No. 3).
• It is true that having a medium such as this makes it easier to use as training materials and a tool for sharing information. It is also fun to create teaching materials (No. 4).

#### 3.2.4. Privacy Protection

• Using patients is ethically difficult. With this method, there is no need for a patient to say they do not want to appear since they do not want their face to be seen (No. 4).
• An actual patient is good if you want realism; however, realism is ethically difficult (No. 1).

### 3.3. Promoting Body Awareness

Responses were classified into three categories as follows: novice nurse training, used for patient guidance (promoting patient understanding and fall prevention), and moves have not been revealed until now.

Participants noted that, until now, they wanted to communicate about physical awareness but could not get it all across as a patient safety manager. This applied to both new and experienced nurses. They expressed the need for educational materials that could help foster friendship within a multidisciplinary team.

Participants also reported wanting to better understand the movements of patients during falls. Although the goal was to help patients understand their movements, nurses also expressed a desire to gain a clearer understanding of their patients’ movements. Upon further reflection, the idea of recognizing the mutual movements between patients and nurses emerged.

Some typical responses are as follows.

#### 3.3.1. Novice Nurse Training

• We can generate interest and curiosity using these media, and learn from these experiences by thinking about things together. Instead of only providing verbal warnings, we could watch it (how the new nurse transforms into an avatar) together, and we can give advice like “Oh, this is it” (No. 3).
• It is a good way to convey details that are not verbalized by making them visible (No. 4).
• I think younger people probably do not have much experience with how older patients move. Indeed, we are currently in the video generation. It is also quite difficult to understand from the text alone. They are people from an era where you see with your eyes, after all. Actually, using avatars or something to experience it would be great (No. 5).
• I think education through text is probably getting quite difficult because people cannot express their body movements (No. 6).
• As a patient safety educator, there are things I would like to also convey to new nurses. Patient safety education is not only for new nurses but also for experienced nurses and various other professions, including medical professionals, administrative staff, and nutritionists; therefore, anyone can participate. This appears particularly useful for training new staff, who come together from various professions, since the goal is for them to become friendly as a team (No. 1).

#### 3.3.2. Used for Patient Guidance

##### 3.3.2.1. Promoting Patient Understanding

• It may be helpful to have the patient watch some exercises. You can use this to get a little idea of what the next movement is, for example, “I am getting up” or “I am standing up.” Well, you explain it verbally, but they do not understand; if they can see it visually, maybe they will understand “Oh, next I am going to stand up” (No. 1).
• The patients are getting older, after all. There are pamphlets and things like that, and the nurses actually show them. However, getting the message across is challenging. They forget, and I think there are cognitive issues. For example, inform the patient in advance about risky limb positions after surgery. It can show the patient what is going to happen next (No. 3).
• If you have patients wear it and move around while using it, you might actually be able to identify where risks exist (No. 6).

##### 3.3.2.2. Fall Prevention

• Patients fall in unexpected ways, do they not? For instance, a sliding door on the side with a handle—the handle is visible to the patient like a handrail, leading them to put their weight on it. However, this risk is usually recognized when they actually fall. Until now, we have never shown such a situation in a video (No. 1).
• If we can look at the posture of patients when they fall, we might say things like, “Oh, they are bumping into things like this” (No. 4).
• Inform them that they cannot lift their feet as much as they think and that they are actually shuffling (No. 5).
• Patients usually fall when putting on their shoes. That is what happens—their upper body moves forward (No. 6).

#### 3.3.3. Moves Have Not Been Revealed Until Now

• If a patient falls in the toilet, how far will the door open? We can open doors properly if we can identify their falling position (No. 1).
• Ah, toilet assistance, etc.—maybe that is why there are so many situations where it can be used (No. 4).
• Check whether the movements of patients and/or nurses are correct. There is a plan to prevent back pain among staff. In Basic Life Support (BLS) training, you might be able to see posture, right? Ah, to evaluate yourself. After all, you might think, “Your elbows are completely bent, which is ineffective when pressing the chest” (No 3).
• The way you move varies slightly depending on the location. When you actually look at it, it might not work at all. Yes, checking the movement objectively is vital (No. 3).
• Both the nurse and patient—I want to know their movements (No. 5).
• It would be good to have teaching materials that allow a comparison between safe and dangerous movements (No. 3).

### 3.4. Patient Safety Using Avatars

Responses were classified into two categories as follows: limitations of this avatar and for risk prediction training. Predicting risks is necessary for patient safety. However, the avatar alone does not clearly portray the situation. Therefore, combining it with the surrounding environment was considered necessary. Some typical responses are as follows.

#### 3.4.1. Limitations of This Avatar

• I can see the movements of the caregiver; however, understanding what they are doing is a little difficult. Can this be used even if two avatars are overlapping? I want to know the movements that show the relationships between multiple people (No. 1).
• If you are going to use only the body movements of a moving person as teaching material, I wonder if it is best to take a picture of the individual and then combine it. I hope they consider the position of the bed and things like that (No. 5).
• It is hard to see because there is no background. It is a little difficult to see what the person is trying to do. Therefore, it might be a little easier to understand if you can see the individual, even if it is a little translucent (No. 6).
• It would be nice if it could sense the back to ensure the detection of the movement of the back (No. 4).

#### 3.4.2. For Risk Prediction Training

• Well, prior training is necessary (No. 1).
• Communicating the perspective of experienced employees to new employees is important. When monitoring patients, new nurses watch the patients to make sure they do not fall. Experienced nurses prepare by predicting that they should clean up here or in a position that will support them if they fall. For example, they put their hand on the side that is likely to hit something that will fall (No. 6).
• They train by looking at pictures on paper, but it is quite difficult to predict, is it not? However, it might be easier for them to understand when it is a video (No. 4).
• In future educational materials, using avatars and being able to see the movements of both the caregiver and patient will make it easier to understand where the risks emerge (No. 5).

## 4. Discussion

### 4.1. Patient Safety Education Using New Technology

This study’s limitations include the small sample size, a restricted simulation setting due to the use of a conference room, and the use of a non‐nursing‐specific motion‐capture tool. Initially, this study began with an avatar experience, which facilitated discussions on patient safety management and education. Rather than exploring profound emotions, the study focused on new experiences and proposing ideas for clinical application. However, the patient safety manager’s suggestions for new methods were significant. The “mocopi” software used a silhouette‐only avatar, and 3D tracking was easy to implement, which was a major advantage. Its human‐like appearance—as shown in Figure 1, rather than an animated character—along with the ability to track movements appear to make it particularly suitable for patient safety education.

Poza‐Méndez et al. [26] demonstrated that social networks have emerged as powerful educational tools, providing new opportunities for interactive learning. Novice nurses typically perceive fewer cues than experienced nurses. However, they learn to observe effectively, obtain necessary information, and prospectively assess patients’ conditions by participating in the simulation education program [27]. In the future, nursing students and practicing nurses will need to acquire the necessary knowledge and skills to effectively evaluate and safely integrate artificial intelligence (AI)–based medical technologies, including virtual avatar applications [28].

Although not without challenges, myriad information and communication technologies (ICTs) can be adopted to support complex and diversified practices and interventions in nursing. Nurse and patient satisfaction or dissatisfaction with ICTs has been frequently reported [29]. However, the avatars used in this study were perceived as easy and enjoyable to use. Such an experience may help reduce resistance to adopting this technology. Therefore, nurses and other clinicians should collaborate with patients and caregivers to ensure that emerging virtual reality and avatar technologies are appropriately designed, evaluated, implemented, and used [30].

Participants in this study emphasized that patient safety education is important not only for new and experienced nurses but also for interprofessional teams. Although the avatars used were not specifically created for nursing, they had limitations. This study highlighted the clear need for patient safety education to share individuals’ movements without revealing their identities.

The nursing workforce comprises multiple generations, each with distinct values and beliefs. Younger nurses are more adaptable to technology and professional development, whereas older nurses prioritize clinical care and patient outcomes [31]. The method used in this study was simple and accessible to all participants and did not reveal who was good or bad at technology. Although the “mocopi” was created for recreational dance, the fact that participants could understand body awareness through the avatar and use new technology garnered positive feedback.

### 4.2. Protecting Privacy and Promoting Body Awareness

Facial expressions are an important focus in nursing care; however, this study deliberately focused solely on body movements. This approach represented a new attempt to demonstrate an individual’s movements without identifying them. By using the avatar with mobile motion capture, nurses can learn about their movements without concern for physical characteristics, such as body shape, clothing, or facial expressions.

The importance of and attention to patient privacy have been directed primarily toward medical data protection via electronic means in recent decades. However, protecting patients’ and doctors’ physical and informational privacy remains essential [32]. The societal implications of AI and robotics extend to accessibility, privacy, and data security, which are paramount concerns [33]. Accordingly, continued research is needed to identify approaches that improve body awareness while protecting privacy.

Remote patient monitoring (RPM) enables the delivery of real‐time clinical patient data to the medical teams. However, additional challenges to its widespread adoption include increased administrative burden for physicians and patient data privacy issues [34]. In this study, monitoring in the toilet, which participants suggested, will also face similar challenges associated with RPM. Therefore, using avatars protects privacy and opens up possibilities for monitoring movements.

Participants indicated a desire to understand not only nurses’ body awareness but also patients’ movements and the interactions between nurses and patients. They suggested that body awareness could be used for self‐evaluation, such as assessing the posture of nurses with back pain or correcting their body movements during BLS. Nurses cannot see through the door to protect patients’ privacy. Therefore, understanding the circumstances of a fall would allow them to communicate the situation to the patient’s family. Nurses could also respond more appropriately if they understood which part of the body was impacted when a patient fell. Implementing 3D tracking capable of distinguishing between the movements of both patients and nurses would enable nurses to determine appropriate patient movement. Therefore, strengthening nurses’ body awareness is crucial to ensuring patient safety.

Until now, filming has been restricted to protect privacy. However, it can be used for safety education through machine learning if movement data are accumulated, highlighting interactions between nurses and patients. Sharing information on nurse and patient movements, which has been unavailable until now, would enable obtaining evidence for future patient safety education. Consequently, demonstrating each patient’s current state and possible risks using avatars would be possible.

Techniques for promoting body awareness present new challenges. Healthcare systems and professionals can harness the potential of AI and robotics while ensuring responsible and ethical integration that benefits patients and upholds the highest ethical standards [33]. Therefore, remaining aware of the ethical aspects associated with the use of new equipment is necessary. Nurses will increasingly be required to understand the limitations of each device and exercise the ability to observe both the mind and body.

### 4.3. Possibilities for Nursing Management Using Avatars

This study demonstrated that patient safety education, such as highlighting hazardous objects on site, cannot be conveyed solely through an avatar. In this context, the avatar was akin to being in a silent movie that requires supplementation with verbal explanations.

The “metaverse” represents the merging of two worlds into an immersive, online, virtual, connected environment where participants actively engage with 3D content and interact through digital avatars. It offers many possibilities and challenges for introducing new methods in nursing [35]. Avatars are also expected to support communication training since they allow individuals to converse as fictional characters who are not identified with the person in question. Therefore, patient safety education using an avatar will potentially be a medium to promote both technical and nontechnical skills.

To date, only a few studies exist on the use of avatars in nursing education. Body awareness education using video has also been limited to the field of surgery. Automated surgical video analysis has been shown to enhance the assessment of nontechnical skills [36]. Simulated practice sessions and self‐evaluating video‐recorded performance have been identified as promising and implementable teaching strategies [37]. As video recording becomes easier and cameras get smaller, the demand for greater privacy protection will likely increase.

AI may be applied in the field of nursing management in the future. Suggestions for improving its adoption in healthcare have been highlighted—enhancing personalization and customizability, as well as improving the empathy and personification of AI‐enabled chatbots and avatars [38]. This study suggests that sharing actions using avatars and encouraging speech through simulations—leveraging the avatar’s inherent characteristics—can contribute to patient safety. Although avatars alone cannot fully convey context, their limitations may encourage reflection and verbalization. Using avatar technology can support the mutual development of both technical and nontechnical skills. This study was exploratory and qualitative; therefore, clarifying body awareness and movement‐based knowledge sharing are expected to contribute to improving the quality of nursing management.

## 5. Conclusion

In this study, participants were interviewed after a new experience of using an avatar. All participants were female and experienced nurses, with no novice nurses. This small‐scale, exploratory qualitative study included six participants. Prior to that, she had experience as a lecturer in information science. In the future, she plans to conduct motion capture of nurses and patients in planned situations, compare their movements, and accumulate the data.

The motion‐capture system was not designed for nursing scenarios and may not fully and accurately reflect such situations. Additionally, the “mocopi” is manufactured in Japan, making it challenging to implement in other countries. However, the technology’s transferability as a body awareness technique for oneself and others, while protecting privacy, can be increased if other systems can be used to eliminate individual characteristics.

To ensure patient safety, nurses must recognize and understand both their own body movements and those of patients. Observation skills may be improved through the use of avatars to remove personal information, after which the linguistic ability to express oneself can be cultivated. Since avatars can be used to convey movements without identifying individuals, mobile motion‐capture‐linked avatars may serve as a new method for patient safety education. The movement‐based knowledge sharing developed through avatar use can contribute to improving nursing management quality.

### 5.1. Implications for Nursing and Health Policy

Until now, obtaining movement‐related information has been challenging due to privacy protection. However, using avatars that eliminate identifiable personal elements can make it easier to obtain movement data for nurses, patients, and their interactions. This approach will allow nurses to be at ease while learning about each movement.

The “mocopi” system is designed to remove the background and retain only the individual’s movements, using a uniform, preset background color. This feature allows photographs or illustrations to be composited into the background as needed. In addition, recent updates to the “mocopi” system have enabled integration with other software, expanding the potential for more flexible and context‐rich applications. Furthermore, adopting technologies that capture movements without jeopardizing an individual’s privacy may enable the accumulation of evidence of body awareness across countries. The quality of nursing management may be further improved through the use of new equipment to enhance nurses’ observation and language skills.

## Funding

This work was supported by JSPS KAKENHI (Grant number 23K02822).

## Disclosure

Some results from this study were presented as posters at an academic conference (The Society of Instrument and Control Engineers, System Integration 2024).

## Ethics Statement

This study was approved by the Ethics Committee of Keio University, Faculty of Nursing and Medical Care (approval no. 334).

## Conflicts of Interest

The author declares no conflicts of interest.

## Supporting Information

Details of the preparation, during the interview, and after the interviews for this study are provided in the Appendix 1.

## Supporting information


**Supporting Information** Additional supporting information can be found online in the Supporting Information section.

## Data Availability

The data that support the findings of this study are available from the corresponding author upon reasonable request.
