# Supplementary material for: A Qualitative Exploratory Study of Patient Safety and Nurses’ Body Awareness Using Motion‐Capture‐Linked Avatars
Source: J Nurs Manag. 2026 Mar 26;2026:9415472. doi: 10.1155/jonm/9415472 (PMC13140177; doi:10.1155/jonm/9415472)
Supplement: Supplementary file 1 — Supporting Information Additional supporting information can be found online in the Supporting Information section. [file JONM-2026-9415472-s001.zip › Appendix_1_2026_1.pdf]

Appendix 1

**1. Preparation**	
Research ethics application	Participant recruitment method/survey content and method/request letter to participants/explanation of participants' implementation details/review of consent forms, among others
Creating an example video	Video creation, no audio<br>1) Video recording of caregiving situations<br>2) Mobile motion-capture using the "mocopi"<br>-Three-dimensional full body tracking<br>-Removal of background using chromakey effect in the video editing software EDIUS10 (Grass Valley K.K., Japan)<br>-Cut out scenes other than those that are necessary, and extract still images to be presented
Recruiting participants	Recruiting participants/arranging schedules/reserving and providing information on conference rooms that are accessible to participants and equipped with wireless LAN
Device	"mocopi"<br>Mobile Motion Capture (Sony)<br>https://www.sony.jp/mocopi/<br>Sensor<br>-Each piece: 3.2 cm diameter, 8 g<br>-The following six locations: the back of the head, lower back, both wrists, and both ankles<br>-Wear it using a special wristband<br>-Measurement method: Acceleration + Angular velocity sensor 3DoF<br>-Power supply: Li-ion<br>-Communication method: Bluetooth LE Version 5.2<br>-Maximum communication distance: 10 m<br>-Frequency band used: 2.4000–2.4835 GHz<br>Hardware and software<br>-The image is visualized and saved as an avatar using a dedicated smartphone or personal computer application.

	-In this study, an iPad mini (5th generation) was used.<br><br>As of January 2026,<br>-Android 11 or later/iOS 15.7.1 or later<br>-Windows 10 (64-bit) or later<br>Video data<br>-File format: MP4<br>-Video format: MPEG-4 AVC/H.264<br>-Audio format: AAC Audio<br>-Resolution:1920 × 1080<br>-Frame rate: 30 fps
**2. During the interview**	
Informed consent	Greetings, self-introduction/explanation and agreement (5 min)
Participant observation and recording	Participant observation, audio recording explanation, and consent<br>Sharing images with participants<br>-Watching actual caregiving scenes (2 min)<br>-Watch a video of a human avatar wearing the “mocopi” in a caregiving situation (2 min)
Implementing a program to experience avatar movements<br>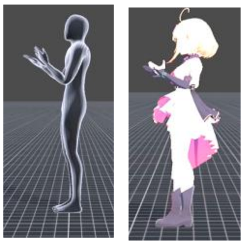	Presentation of instructions and the “mocopi” experience (30 min)<br>-Indication of the location of attachment<br>-Enter your height (in 5 cm increments)<br>-Connection between the center and iPad<br>While standing, give the signal and take one step forward<br>-Avatar selection (left)<br>-Start recording as an avatar
Group interview	Interview guide (20 min)<br>1. What are your honest thoughts about the sample video and your avatar experience? (Usability of the avatar)<br>2. Is it necessary to share still images and videos? What kind of educational materials will be effective? How do you educate nurses and patients about body movement? (Body awareness)<br>3. What educational materials do you and your patients need to ensure patient safety?

	4. What type of educational materials do you need to raise awareness of patient safety? (For learning hazard prediction)
Answers to the questionnaire	Writing on paper (10 min)<br>Age/Sex/Years of nursing experience/Years of experience as a medical safety manager/ and Prior experience using an avatar<br>- Calculated using Microsoft Excel<br>The same questions as in the group interview were asked in an open-ended format.
Debriefing time	
**3. After the interview**	
Creating a verbatim transcript	-Writing research notes<br>-Transcription of recorded data
Coding	-Data conversion of responses<br>-Categorization<br>-Category naming<br>-Saturation of data into categories<br>-Ensuring the reliability of the categories formed
Final themes	-Clarifying the theme<br>-Category 3 sub-category (Table 1)
Clarifying the limitations of this study	Limitations of this study<br>-Uniformity of participants<br>-Small number<br>-Coding by one researcher
